# WDR72 Drives Esophageal Squamous Cell Carcinoma Progression by Inhibiting Autophagy via the PI3K/Akt/mTOR Pathway

**DOI:** 10.1002/kjm2.70235

**Published:** 2026-06-01

**Authors:** Hao Wu, Zhong‐Xiang Jiang, Zheng Jiang

**Affiliations:** ^1^ Department of Gastroenterology The First Affiliated Hospital of Chongqing Medical University Chongqing China; ^2^ Chongqing General Hospital (Chongqing General Hospital, Chongqing University) Chongqing China; ^3^ Department of Respiratory and Critical Care Medicine Chongqing General Hospital (Chongqing General Hospital, Chongqing University) Chongqing China

**Keywords:** autophagy, esophageal cancer, PI3K/Akt/mTOR pathway, WDR72

## Abstract

Esophageal squamous cell carcinoma (ESCC) is an aggressive malignancy with a high rate of recurrence and metastasis, necessitating the identification of novel therapeutic targets. WD repeat‐containing protein 72 (WDR72) has been linked to various cancers, but its specific biological function and underlying mechanism in ESCC remain largely unexplored. Herein, we investigated whether WDR72 promotes ESCC progression by suppressing autophagy via the PI3K/Akt/mTOR pathway. We first analyzed WDR72 expression using GEO (GSE213565) and validated using the GEPIA public databases. WDR72 mRNA/protein levels were then examined in patient‐derived ESCC tissues and cell lines (EC109, KYSE150). Loss‐ and gain‐of‐function models (shRNA knockdown and pcDNA3.1‐WDR72 overexpression) were used to evaluate proliferation, clonogenicity, apoptosis, migration/invasion, and autophagy. PI3K/Akt/mTOR activity was examined by measuring PI3K, p‐Akt, and p‐mTOR, with pathway dependence tested using the dual PI3K/mTOR inhibitor BEZ235. In vivo tumor growth and metastasis were analyzed in xenograft and lung metastasis models. We found that WDR72 was significantly overexpressed in ESCC tissues and cell lines. WDR72 depletion suppressed malignant phenotypes in vitro and substantially inhibited tumor growth and metastasis in vivo. Mechanistically, WDR72 overexpression activated the PI3K/Akt/mTOR pathway (indicated by elevated p‐Akt and p‐mTOR levels) and concurrently suppressed autophagy (evidenced by decreased Beclin‐1 and LC3‐II/LC3‐I ratio, and increased P62). Notably, the PI3K/mTOR inhibitor BEZ235 abolished the pro‐malignant effects and reversed the autophagy suppression induced by WDR72 overexpression. Collectively, our findings establish that WDR72 acts as an oncogene in ESCC by promoting proliferation, survival, and metastasis via activating the PI3K/Akt/mTOR signaling to suppress autophagy.

## 1 Introduction

Esophageal cancer (EC) ranks among the most common gastrointestinal malignancies worldwide [1]. The predominant histological subtype is esophageal squamous cell carcinoma (ESCC), which arises mainly in the upper and middle regions of the esophagus and accounts for more than 80% of EC cases [2, 3]. Smoking and excessive alcohol consumption are recognized as major risk factors for ESCC [4]. Because early‐stage ESCC often lacks overt symptoms and reliable biomarkers remain scarce, a substantial proportion of patients, often around half, are diagnosed at intermediate to advanced stages [5]. The standard therapeutic modalities for ESCC include radical esophagectomy, chemotherapy and radiotherapy [6]; however, postoperative metastasis or recurrence remains a major hurdle to curative treatment [7]. Indeed, ESCC frequently metastasizes to the liver, lungs and bone, contributing to poor survival outcomes [8]. Therefore, identifying novel molecular biomarkers and therapeutic targets is urgently needed to improve diagnostic precision and treatment efficacy for ESCC.

WD repeat‐containing protein 72 (WDR72) is a member of the WD40‐repeat domain superfamily that functions as a scaffolding protein lacking intrinsic enzymatic activity [9]. Germline mutations in WDR72 are causally associated with autosomal‐recessive amelogenesis imperfecta, in which defective enamel‐forming ameloblasts exhibit retention of enamel matrix proteins and impaired mineralization [10]. Structurally, WDR72 forms β‐propeller‐like blades typical of WD‐repeat proteins, which mediate multiprotein interactions involved in cell‐cycle regulation, vesicle trafficking, and intracellular signal transduction [11]. Emerging evidence has linked WDR72 to cancer pathophysiology. For instance, WDR72 up‐regulation suppresses proliferation and invasion of renal cell carcinoma and is proposed as a prognostic biomarker [12]. Conversely, in lung cancer, WDR72 expression is markedly increased, where it enhances cancer cell stemness by activating the AKT signaling pathway and reshaping the tumor microenvironment through immune‐cell infiltration [13, 14]. Notably, a transcriptomic analysis predicted that WDR72 is significantly overexpressed in EC tissues and may serve as a potential diagnostic indicator [15]. Nevertheless, the precise biological functions and mechanistic roles of WDR72 in ESCC remain unclear, and its involvement in oncogenic signaling pathways has yet to be elucidated.

Among the diverse intracellular cascades driving tumorigenesis, the phosphatidylinositol‐3‐kinase (PI3K)/Akt/mammalian target of rapamycin (mTOR) signaling pathway is one of the most frequently dysregulated and critically regulates cancer cell growth, metabolism, and survival [16, 17]. Activated by receptor tyrosine kinases or oncogenic mutations, PI3K catalyzes the conversion of phosphatidylinositol‐4,5‐bisphosphate (PIP_2_) to phosphatidylinositol‐3,4, 5‐trisphosphate (PIP_3_), which recruits Akt to the plasma membrane where it is phosphorylated and activated. Akt subsequently stimulates mTOR complex 1 (mTORC1) to promote protein synthesis and cell proliferation while concurrently suppressing autophagy [18]. Persistent activation of the PI3K/Akt/mTOR axis is a hallmark of numerous cancers, including breast, colorectal, lung, and esophageal carcinomas [17, 19]. In ESCC, aberrant activation of this pathway correlates with increased tumor invasiveness, chemoresistance, and poor clinical outcomes [20, 21]. Furthermore, because mTOR serves as a central negative regulator of autophagy, hyperactivation of the PI3K/Akt/mTOR cascade can inhibit autophagic flux, enabling malignant cells to evade stress‐induced death and sustain oncogenic metabolism [22, 23]. Consequently, modulation of autophagy through targeting the PI3K/Akt/mTOR pathway has attracted growing attention as a therapeutic approach in ESCC.

Given the previously documented relationship between WDR72 and the Akt signaling in lung cancer [13], and the central role of the PI3K/Akt/mTOR pathway in regulating cell survival and autophagy [18], it is plausible that WDR72 may promote ESCC development through this oncogenic axis. However, the specific relationship between WDR72 and PI3K/Akt/mTOR signaling and their combined impact on autophagy in ESCC remains unknown. Therefore, the present study was undertaken to elucidate the biological function and molecular mechanism of WDR72 in ESCC, focusing particularly on whether WDR72 inhibits autophagy via activation of the PI3K/Akt/mTOR pathway to promote tumor progression. Clarifying this mechanism will not only enhance understanding of ESCC pathogenesis but may also identify WDR72 as a promising biomarker and therapeutic target for clinical intervention.

## 2 Materials and Methods

### 2.1 Bioinformatics Analysis

Publicly available gene expression datasets related to ESCC were obtained from the Gene Expression Omnibus (GEO) database (https://www.ncbi.nlm.nih.gov/geo/). Datasets were selected based on the following criteria: (i) inclusion of both ESCC and adjacent nontumorous esophageal tissues; (ii) sufficient sample size and biological replicates; and (iii) comparable microarray or RNA‐sequencing platforms to ensure data consistency. The dataset GSE213565 was used for differential expression analysis. Raw expression data were preprocessed and normalized using the “limma” R package (version 3.54.0; Bioconductor, USA). Differentially expressed genes (DEGs) between ESCC and normal tissues were identified using empirical Bayes moderation with a threshold of |log_2_ fold change| ≥ 1 and adjusted *p* < 0.05. The “ggplot2” and “pheatmap” packages (version 3.4.2 and 1.0.12, respectively; CRAN, USA) were applied to visualize the results as volcano plots and clustered heatmaps.

Expression levels of WDR72 in ESCC were further validated using the Gene Expression Profiling Interactive Analysis (GEPIA) database (http://gepia.cancer‐pku.cn/), which integrates data from The Cancer Genome Atlas (TCGA) and Genotype‐Tissue Expression (GTEx) projects. Comparative analyses between tumor and normal esophageal tissues were performed to confirm differential WDR72 expression across independent datasets. All analyses were conducted in R software (version 4.3.0; R Foundation for Statistical Computing, Vienna, Austria).

### 2.2 Patient Samples

Human ESCC tissues and matched adjacent nontumorous esophageal mucosa were collected from 15 patients who underwent surgical resection at the First Affiliated Hospital of Chongqing Medical University (Chongqing, China) between 2022 and 2024. None of the patients had received preoperative chemotherapy, radiotherapy, or immunotherapy prior to surgery. The diagnosis of ESCC was histopathologically confirmed by two independent pathologists according to the World Health Organization (WHO) classification of digestive system tumors (2019 edition) [24].

Clinicopathological characteristics, including patient age, sex, tumor size, histological grade, TNM stage, and lymph node metastasis, were obtained from hospital medical records. Only patients with complete clinical data and without concurrent malignancies, autoimmune disorders, or severe infections were included in this study to minimize confounding effects.

Immediately after surgical resection, tumor and paired adjacent normal tissues (located at least 5 cm away from the tumor margin) were snap‐frozen in liquid nitrogen and stored at −80°C until RNA and protein extraction. All patients provided written informed consent prior to participation. The study protocol was reviewed and approved by the Ethics Committee of our hospital, and all procedures were conducted in accordance with the Declaration of Helsinki.

### 2.3 Cell Culture

Human ESCC cell lines EC109 and KYSE150, as well as the normal human esophageal epithelial cell line (HEEC), were obtained from the Shanghai Institute of Biochemistry and Cell Biology (SIBCB, Shanghai, China). All cell lines were authenticated by short tandem repeat (STR) profiling and routinely tested to confirm the absence of Mycoplasma contamination before use. Cells were cultured in RPMI‐1640 medium (Gibco, Thermo Fisher Scientific, USA) supplemented with 10% fetal bovine serum (FBS; Gibco, USA), 100 U/mL penicillin, and 100 μg/mL streptomycin (Solarbio, China). Cultures were maintained at 37°C in a humidified atmosphere containing 5% CO_2_.

For pharmacological inhibition experiments, cells were treated with 50 nM BEZ235 (a dual PI3K/mTOR inhibitor; MedChemExpress, USA; Cat. No. HY‐50673) or 100 μM chloroquine (a lysosomal inhibitor; MedChemExpress; Cat. No. C6628) for 24 h, while control cells received an equal volume of vehicle (0.1% DMSO).

### 2.4 Cell Transfection

EC109 and KYSE150 cells were seeded in 6‐well plates at a density of 6 × 10^5^ cells/well and allowed to reach approximately 70% confluence before transfection. The WDR72 overexpression plasmid was constructed by cloning the full‐length human WDR72 cDNA into the pcDNA3.1 vector (Geenseed Biotech, Guangzhou, China); the corresponding empty vector served as a negative control (pcDNA‐NC). For gene silencing, short hairpin RNA (shRNA) sequences targeting WDR72 (sh‐WDR72) and AKT (sh‐AKT) and their corresponding nontargeting control (sh‐NC) were synthesized by GeneChem (Shanghai, China).

Transfections were performed using Lipofectamine 3000 reagent (Invitrogen, USA) following the manufacturer's instructions. After 48 h of transfection, cells were harvested for subsequent assays, and transfection efficiency was confirmed by quantitative real‐time PCR (qRT‐PCR) and Western blot analysis.

### 2.5 Reverse Transcription and Quantitative Real‐Time PCR (RT‐qPCR)

Total RNA was isolated from ESCC tissues or cultured cells using TRIzol reagent (Invitrogen, Thermo Fisher Scientific, USA) following the manufacturer's protocol. RNA purity and concentration were determined using a NanoDrop 2000 spectrophotometer (Thermo Fisher Scientific, USA), and samples with an A260/A280 ratio between 1.8 and 2.0 were used for further analysis. Complementary DNA (cDNA) was synthesized from 1 μg of total RNA using the PrimeScript RT Reagent Kit (TaKaRa, Dalian, China) according to the manufacturer's instructions. Quantitative real‐time PCR was performed using TB Green Premix Ex Taq II (TaKaRa, Dalian, China) on a QuantStudio 5 Real‐Time PCR System (Applied Biosystems, USA). Relative mRNA expression levels were calculated using the 2^−ΔΔ*Ct*^ method, with GAPDH serving as the internal control.

### 2.6 Western Blot Analysis

Total cellular or tissue proteins were extracted using RIPA lysis buffer (Beyotime, Shanghai, China). Protein concentrations were quantified using the BCA Protein Assay Kit (Thermo Fisher Scientific, USA). Equal amounts of protein (20 μg) were separated by 12% SDS‐polyacrylamide gel electrophoresis and transferred onto polyvinylidene difluoride membranes (Millipore, USA). Membranes were blocked with 5% nonfat milk in TBST buffer (20 mM Tris‐HCl, 150 mM NaCl, 0.1% Tween‐20, pH 7.5) for 2 h at room temperature, followed by incubation overnight at 4°C with primary antibodies against WDR72, Bax, Bcl‐2, cleaved caspase‐3, Beclin‐1, LC3‐I/II, p62, PI3K, AKT, phospho‐AKT (Ser473), mTOR, phospho‐mTOR (Ser2448), β‐actin, and GAPDH (all from Abcam, Cambridge, UK). After washing, membranes were incubated with HRP‐conjugated goat anti‐rabbit or anti‐mouse secondary antibodies (Abcam, UK) for 2 h at room temperature. Protein bands were visualized using an enhanced chemiluminescence (ECL) detection kit (Thermo Fisher Scientific, USA) and quantified by ImageJ software (NIH, USA).

### 2.7 Cell Counting Kit‐8 (CCK‐8) Viability Assay

Cell viability was evaluated using the CCK‐8 (Dojindo Laboratories, Kumamoto, Japan; Cat. No. CK04) according to the manufacturer's instructions. Briefly, EC109 and KYSE150 cells were seeded into 96‐well plates at a density of 2 × 10^3^ cells per well and transfected with the indicated plasmids or treated as described. At 0, 24, 48, and 72 h post‐transfection, 10 μL of CCK‐8 reagent was added to each well, followed by incubation for 2 h at 37°C in a humidified incubator containing 5% CO_2_. The absorbance was measured at 450 nm using a microplate reader (Bio‐Rad Laboratories, USA; Model iMark).

### 2.8 Colony Formation Assay

The clonogenic capacity of ESCC cells was evaluated using a colony formation assay. Briefly, EC109 and KYSE150 cells were seeded into 6‐well plates at a density of 500 cells per well and cultured for approximately 10–14 days until visible colonies formed. The culture medium (RPMI‐1640 with 10% FBS; Gibco, USA) was replaced every 3 days. At the endpoint, colonies were gently washed with phosphate‐buffered saline (PBS), fixed with 4% paraformaldehyde (PFA; Solarbio, Beijing, China) for 20–30 min, and stained with 0.1% crystal violet solution (Beyotime, Shanghai, China) for 5–10 min at room temperature. After washing and air‐drying, colonies containing more than 50 cells were counted under a light microscope (Olympus, Japan).

### 2.9 Flow Cytometry Analysis

Cell apoptosis was evaluated using the Annexin V‐FITC/propidium iodide (PI) Apoptosis Detection Kit (BD Biosciences, San Jose, CA, USA) according to the manufacturer's instructions. Briefly, EC109 and KYSE150 cells were collected by trypsinization without EDTA, washed twice with ice‐cold PBS, and resuspended in 1× binding buffer at a concentration of 1 × 10^6^ cells/mL. Subsequently, 5 μL Annexin V‐FITC and 5 μL PI were added to 100 μL of the cell suspension and incubated for 15 min at room temperature in the dark. After staining, 400 μL binding buffer was added, and apoptotic cells were immediately analyzed using a flow cytometer (FACSCalibur, BD Biosciences, USA). Data were processed and quantified using FlowJo software (version 10.8.1; Tree Star, USA).

### 2.10 Transwell Invasion Assay

Cell invasive ability was evaluated using Transwell chambers with 8.0‐μm pore polycarbonate membranes (Corning Costar, USA) precoated with Matrigel (BD Biosciences, USA) to simulate the extracellular matrix. Briefly, 1 × 10^5^ EC109 and KYSE150 cells were suspended in 200 μL serum‐free RPMI‐1640 medium and seeded into the upper chamber, while 600 μL of complete medium containing 10% FBS was added to the lower chamber as a chemoattractant. After incubation for 24 h at 37°C, noninvading cells on the upper surface were gently removed with a cotton swab. The invaded cells on the lower surface were fixed with 4% paraformaldehyde (Solarbio, Beijing, China) for 20 min and stained with 0.1% crystal violet solution (Beyotime, Shanghai, China) for 5–10 min. Stained cells were imaged under an inverted fluorescence microscope (Olympus, Tokyo, Japan) and counted in five randomly selected fields per membrane.

### 2.11 Wound‐Healing Assay

Cell migratory ability was assessed using a wound‐healing assay. Briefly, EC109 and KYSE150 cells were seeded into 6‐well plates and cultured until they reached approximately 90% confluence. A linear scratch was created across the monolayer using a 200‐μL sterile pipette tip, after which detached cells were removed by washing twice with PBS. Cells were then incubated in serum‐free RPMI‐1640 medium (Gibco, Thermo Fisher Scientific, USA) for 24 h to minimize proliferation‐related effects. Images of the wound area were captured at 0 and 24 h using an inverted microscope (Olympus, Tokyo, Japan). The wound closure rate was quantified using ImageJ software (NIH, USA).

### 2.12 Immunofluorescence (IF) Staining

EC109 and KYSE150 cells were seeded onto sterile glass coverslips and fixed with 4% paraformaldehyde (PFA; Solarbio, Beijing, China) for 10 min at room temperature. Cells were then permeabilized with 0.5% Triton X‐100 (Beyotime, Shanghai, China) for 30 min, followed by blocking with 5% bovine serum albumin (BSA; Sigma‐Aldrich, USA) for 1 h to reduce nonspecific binding. Subsequently, cells were incubated overnight at 4°C with the indicated primary antibodies (anti‐LC3B, anti‐Beclin‐1, anti‐WDR72; Abcam, Cambridge, UK). After washing three times with PBS, cells were incubated with appropriate Alexa Fluor‐conjugated secondary antibodies (Abcam, UK) for 1 h at room temperature in the dark. Nuclei were counterstained with 4′,6‐diamidino‐2‐phenylindole (DAPI; Beyotime, China) for 5 min. Fluorescence images were captured using a confocal laser scanning microscope (Leica TCS SP8, Wetzlar, Germany), and fluorescence intensity was quantified using ImageJ software (NIH, USA).

### 2.13 Animal Experiments

All animal procedures were approved by the Ethics Committee of our hospital and conducted in accordance with the ARRIVE guidelines. Male BALB/c nude mice (4–6 weeks old; 18–22 g) were purchased from the Experimental Animal Center of the First Affiliated Hospital of Chongqing Medical University (Chongqing, China) and housed under specific pathogen‐free (SPF) conditions (22°C ± 2°C, 55% ± 5% humidity, 12 h light/dark cycle) with ad libitum access to food and water.

For xenograft tumor formation, mice were randomly assigned into three groups (*n* = 5 per group): control, sh‐NC, and sh‐WDR72. EC109 cells (5 × 10^6^) stably transfected with sh‐WDR72 or sh‐NC were suspended in 100 μL PBS and subcutaneously injected into the right flank of each mouse. Tumor volumes were measured every 5 days using digital calipers and calculated as (length × width^2^)/2. After 30 days, mice were anesthetized with 2% isoflurane (RWD Life Science, Shenzhen, China) and euthanized by cervical dislocation.

For the experimental metastasis assay, EC109 cells transfected with sh‐WDR72 or sh‐NC were injected into the tail vein (1 × 10^6^ cells in 100 μL PBS) of nude mice. After 5 weeks, mice were injected intraperitoneally with d‐luciferin (30 mg/kg; Sigma‐Aldrich, USA; Cat. No. L9504), anesthetized, and imaged using the IVIS Spectrum in vivo imaging system (Caliper Life Sciences, Hopkinton, MA, USA). Metastatic nodules were quantified, and tumor tissues were collected for Western blotting.

### 2.14 Statistical Analysis

All statistical analyses were performed using GraphPad Prism software (version 8.0; GraphPad Software Inc., San Diego, CA, USA). Data are presented as the mean ± standard deviation (SD) from at least three independent experiments. Comparisons between two groups were conducted using an unpaired two‐tailed Student's *t*‐test, whereas comparisons among multiple groups were analyzed by one‐way analysis of variance (ANOVA) followed by Tukey's post hoc test. A *p*‐value of < 0.05 was considered statistically significant.

## 3 Results

### 3.1 WDR72 Is Overexpressed in ESCC

**FIGURE 1:**
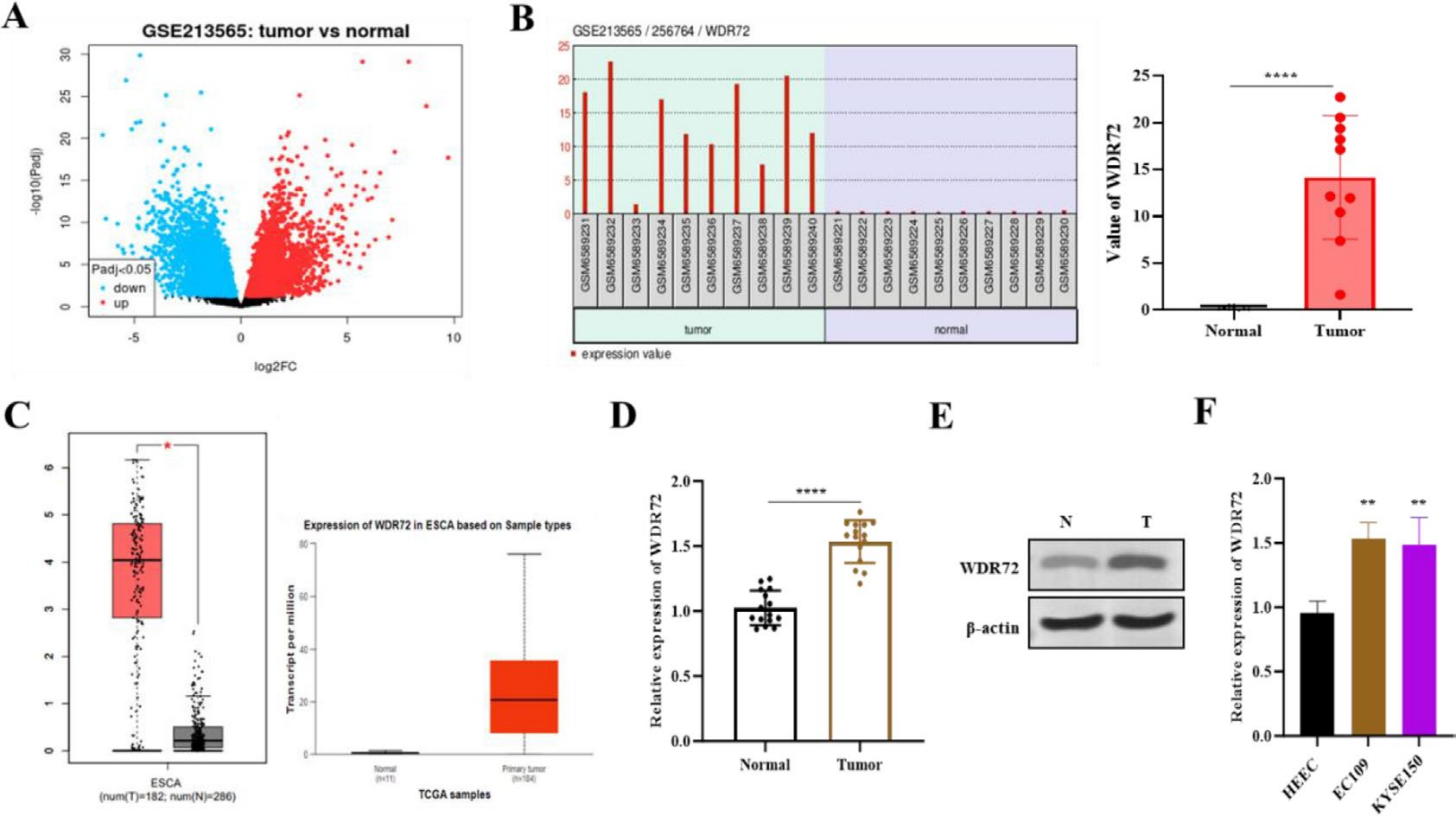
WDR72 is highly expressed in esophageal squamous cell carcinoma (ESCC). (A) Volcano plot showing differentially expressed genes (DEGs) in the GSE213565 dataset, with significantly upregulated and downregulated genes highlighted. (B) WDR72 expression levels in ESCC and adjacent normal tissues analyzed from the GSE213565 dataset. (C) GEPIA database analysis confirming elevated WDR72 expression in esophageal carcinoma (ESCA) tissues compared with normal esophageal tissues. (D‐E) Quantitative RT‐PCR and Western blot validation of WDR72 mRNA and protein expression in paired tumor and adjacent normal tissues from ESCC patients (*n* = 15). (F) RT‐qPCR analysis of WDR72 mRNA expression in the normal esophageal epithelial cell line (HEEC) and ESCC cell lines (EC109 and KYSE150). Data represent the mean ± SD from three independent experiments. Statistical analysis was performed using an unpaired two‐tailed t‐test. *p < 0.05, **p < 0.01, ****p < 0.0001.

To identify potential oncogenic drivers of ESCC, we first performed differentially expressed gene (DEG) analysis using the GEO dataset GSE213565 with thresholds of |log_2_FC| > 1 and *p* < 0.05. As shown in the volcano plot (Figure 1A), several genes were aberrantly expressed in ESCC tissues. Among them, WDR72 was markedly upregulated in ESCC samples compared with adjacent normal tissues based on GSE213565 data (Figure 1B). Consistently, analysis of the GEPIA database confirmed a significant increase in WDR72 expression in esophageal carcinoma (ESCA) tissues relative to normal controls (Figure 1C). To further validate these findings, we examined WDR72 levels in tumor and paired adjacent nontumor tissues from ESCC patients. Both RT‐qPCR and Western blot analyses demonstrated significantly elevated WDR72 mRNA and protein expression in tumor tissues compared with normal tissues (Figure 1D,E). Similarly, ESCC cell lines (EC109 and KYSE150) exhibited higher WDR72 expression than normal esophageal epithelial cells (HEEC) (Figure 1F). These findings collectively confirm that WDR72 is significantly overexpressed in ESCC, suggesting its potential involvement in ESCC progression.

### 3.2 WDR72 Depletion Restrains the Cellular Malignant Phenotypes of ESCC

**FIGURE 2:**
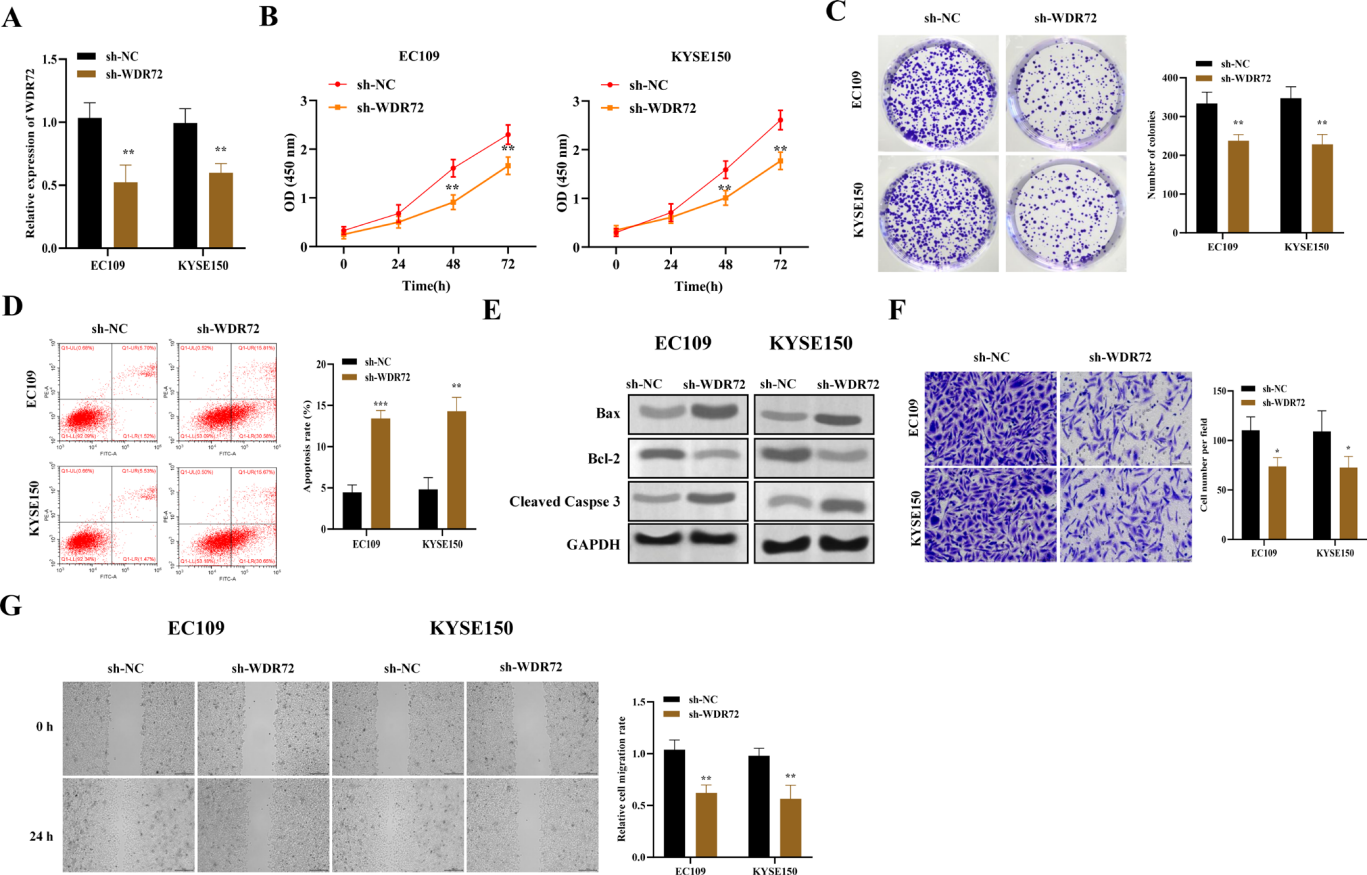
WDR72 depletion suppresses ESCC cell proliferation, migration, and invasion, and induces apoptosis. (A) RT‐qPCR validation of WDR72 knockdown efficiency following transfection with sh‐WDR72 or sh‐NC plasmids in EC109 and KYSE150 cells. (B) Cell viability determined by CCK‐8 assay following WDR72 silencing. (C) Colony formation assay showing reduced clonogenic capacity after WDR72 knockdown. (D) Flow cytometric quantification of apoptotic cells in control and sh‐WDR72‐transfected ESCC cells. (E) Western blot detection of apoptosis‐related proteins (Bax, Bcl‐2, and cleaved caspase‐3) following WDR72 depletion. (F‐G) Transwell and wound‐healing assays assessing the migratory and invasive capabilities of ESCC cells after WDR72 knockdown. Data are shown as the mean ± SD of three independent experiments and analyzed using a two‐tailed unpaired t‐test. *p < 0.05, **p < 0.01, ***p < 0.001.

Given the observed upregulation of WDR72 in ESCC, we next investigated whether WDR72 contributes to the malignant behaviors of ESCC cells in vitro using loss‐of‐function assays. We first successfully silenced endogenous WDR72 expression in EC109 and KYSE150 cells by transfecting specific short hairpin RNA plasmids (sh‐WDR72) (Figure 2A). CCK‐8 assays revealed that WDR72 knockdown significantly reduced cell viability compared with the sh‐NC group (Figure 2B). Similarly, colony formation assays showed a marked reduction in colony numbers in WDR72‐silenced cells (Figure 2C), indicating impaired proliferative capacity. Furthermore, flow cytometry analysis demonstrated that the apoptotic rate was substantially increased upon WDR72 depletion (Figure 2D). Western blotting confirmed that pro‐apoptotic proteins Bax and cleaved caspase‐3 were upregulated, while anti‐apoptotic Bcl‐2 was downregulated following WDR72 silencing (Figure 2E). Moreover, Transwell and wound‐healing assays showed that WDR72 knockdown markedly inhibited the migratory and invasive abilities of ESCC cells (Figure 2F,G). Taken together, these results demonstrate that WDR72 depletion suppresses ESCC cell proliferation, migration, and invasion, while enhancing apoptosis, supporting a tumor‐promoting role of WDR72 in ESCC.

### 3.3 WDR72 Regulates Autophagy in ESCC Cells

**FIGURE 3:**
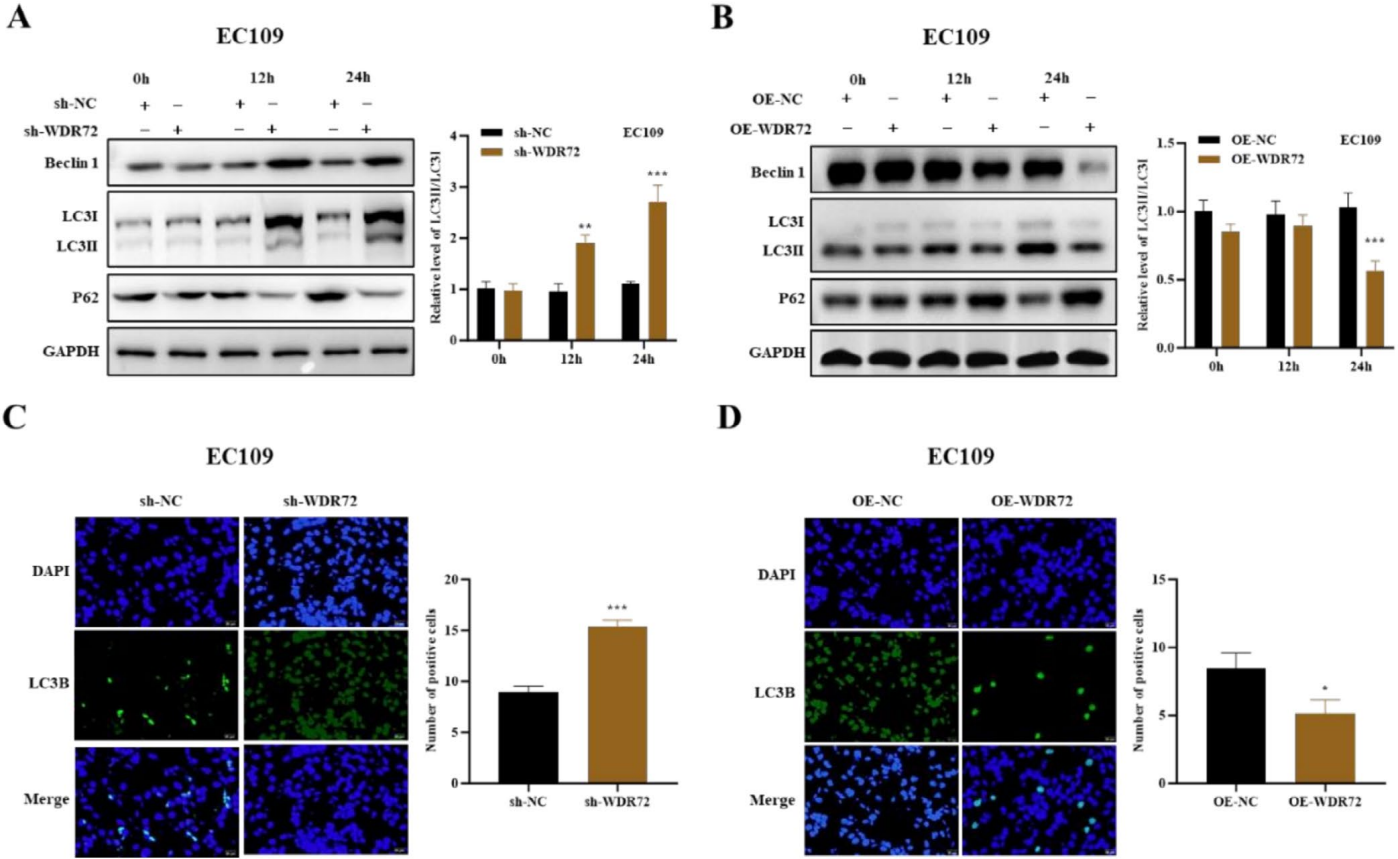
WDR72 modulates autophagy in ESCC cells. (A‐B) Western blot analysis of Beclin‐1 and p62 protein levels in EC109 cells after WDR72 knockdown or overexpression. (C–D) Immunofluorescence (IF) staining of LC3B puncta in EC109 cells after WDR72 silencing or overexpression, showing changes in autophagosome formation. Data are shown as the mean ± SD of three independent experiments and analyzed using a two‐tailed unpaired *t*‐test. *p < 0.05, **p < 0.01, ***p < 0.001.

Because WDR72 depletion promotes apoptosis and that autophagy is a vital process involved in modulating tumor cell survival and stress response [25], we next explored whether WDR72 regulates autophagy in ESCC cells. We constructed both WDR72‐knockdown cells (sh‐WDR72 in EC109) and WDR72‐overexpression cells (pcDNA3.1‐WDR72 in EC109) to examine the impact of both loss and gain of function. Western blot analysis showed that WDR72 knockdown significantly enhanced the levels of the autophagy initiation marker Beclin and reduced the levels of the degradation substrate P62 (also known as SQSTM), indicative of promoted autophagic flux (Figure 3A). Conversely, WDR72 overexpression resulted in the opposite effect, reducing Beclin1 levels while enhancing P62 levels (Figure 3B). Additionally, immunofluorescence staining for LC3B puncta, a structural marker of the autophagosome, showed that the number of LC3B puncta was enhanced by WDR72 deficiency but reduced by WDR72 upregulation (Figure 3C,D). To further confirm whether WDR72 regulates autophagic flux, WDR72‐knockdown cells were treated with the lysosomal inhibitor chloroquine. Western blot analysis demonstrated that chloroquine treatment in WDR72‐deficient cells reduced Beclin1 expression and LC3‐II/I turnover while increasing P62 accumulation, indicating effective inhibition of autophagic flux (Supplementary Figure S1A). Moreover, immunofluorescence staining showed that chloroquine treatment significantly reduced the number of LC3B‐positive EC109 cells following WDR72 knockdown (Figure S1B). These findings collectively demonstrate that WDR72 functions as a negative regulator of autophagy in ESCC cells.

### 3.4 WDR72 Facilitates ESCC Progression Through Modulating the PI3K/AKT/mTOR Pathway

**FIGURE 4:**
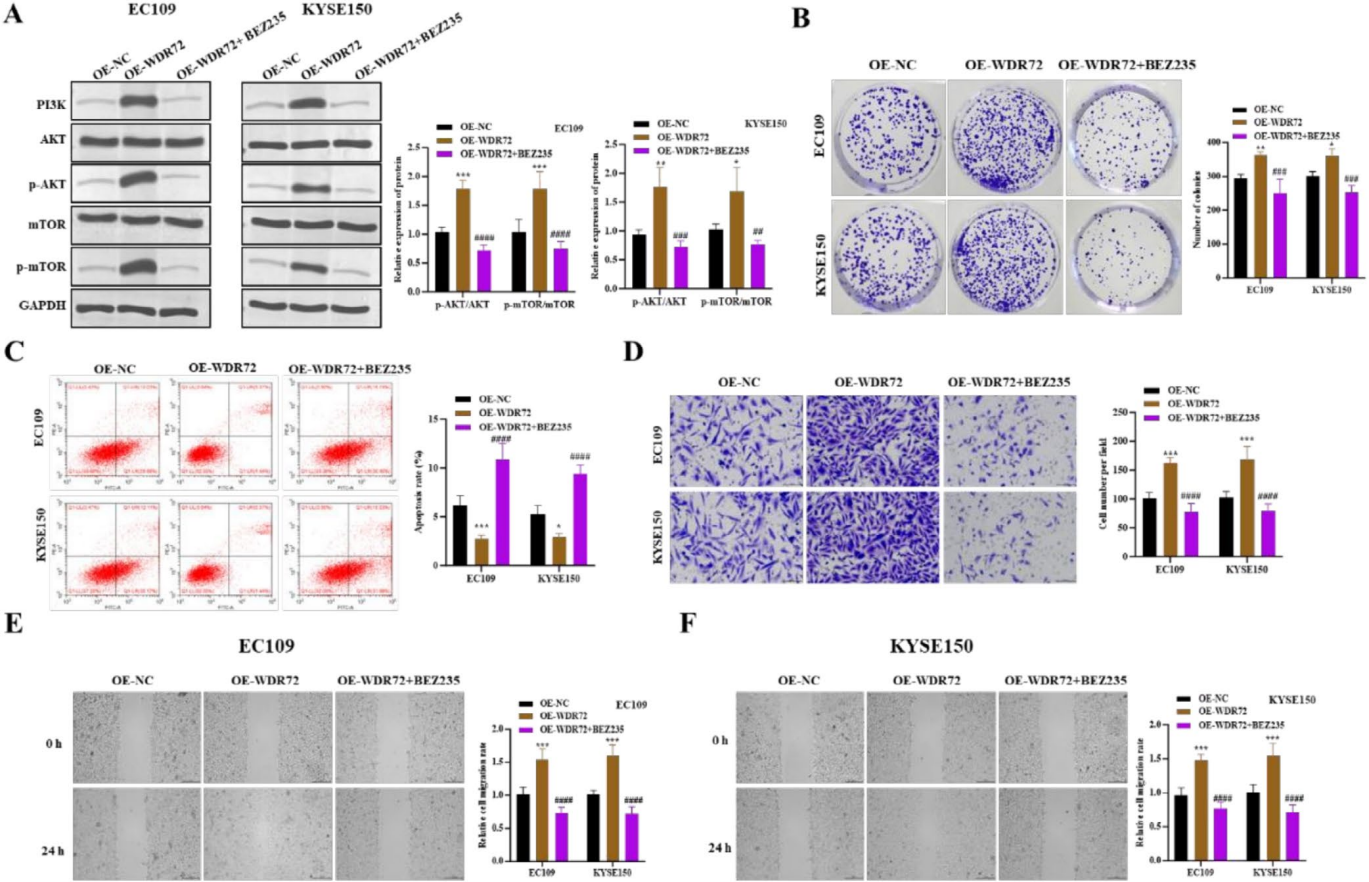
WDR72 promotes ESCC progression through activation of the PI3K/Akt/mTOR signaling pathway. (A) Western blot analysis of PI3K, AKT, p‐AKT, mTOR, and p‐mTOR protein levels in EC109 cells from the control (OE‐NC), WDR72‐overexpressing (OE‐WDR72), and OE‐WDR72 + BEZ235 (PI3K/mTOR inhibitor) groups. (B) Colony formation assay assessing the proliferative ability of cells in the indicated groups. (C) Flow cytometric analysis of apoptosis in the indicated groups. (D–F) Transwell and wound‐healing assays evaluating cell migration and invasion following WDR72 overexpression and BEZ235 treatment. Data are shown as the mean ± SD of three independent experiments and analyzed using one‐way ANOVA followed by Tukey's post hoc test. *p < 0.05, **p < 0.01, ***p < 0.001; ^##^ *p* < 0.01, ^###^ *p* < 0.001, ^####^ *p* < 0.0001.

Given that the PI3K/AKT/mTOR signaling pathway is a key regulator of autophagy and tumor cell survival [17], we next examined whether WDR72 mediates ESCC progression through this pathway. Bioinformatics analysis using the GEPIA database (http://gepia.cancer‐pku.cn/) revealed a significant positive correlation between WDR72 and PIK3CA expression in ESCC samples (Figure S2A), suggesting a potential regulatory association. Therefore, we next explore whether WDR72 regulates this signaling axis. Western blot analysis showed that WDR72 overexpression led to a substantial increase in PI3K, p‐Akt, and p‐mTOR levels. Notably, treatment with BEZ235, a dual PI3K/mTOR inhibitor, markedly reversed these effects (Figure 4A), indicating that WDR72 activates the PI3K/Akt/mTOR pathway. To confirm that the pro‐malignant function of WDR72 is dependent on this pathway activation, we performed rescue experiments using BEZ235 in WDR72‐overexpressing cells. We demonstrated that the increase in colony formation caused by WDR72 upregulation was significantly suppressed after BEZ235 treatment (Figure 4B). Similarly, the reduction in cell apoptosis observed with WDR72 overexpression was abolished and reversed after BEZ235 administration (Figure 4C). Furthermore, BEZ235 treatment prominently attenuated the promoting effect of WDR72 overexpression on cell migratory and invasive capabilities (Figure 4D–F). To further clarify the downstream signaling hierarchy, additional rescue experiments were performed using AKT knockdown in WDR72‐overexpressing cells. Western blot analysis demonstrated that AKT depletion reversed the effects of WDR72 overexpression, resulting in increased Beclin1 expression, an elevated LC3‐II/LC3‐I ratio, and decreased P62 levels. Furthermore, immunofluorescence staining confirmed that AKT knockdown restored LC3B puncta formation that had been suppressed by WDR72 overexpression (Figure S2B,C). Collectively, these findings demonstrate that WDR72 promotes ESCC proliferation, migration, and invasion through activation of the PI3K/AKT/mTOR signaling pathway.

### 3.5 WDR72 Suppresses Autophagy in ESCC Through the PI3K/Akt/mTOR Pathway

**FIGURE 5:**
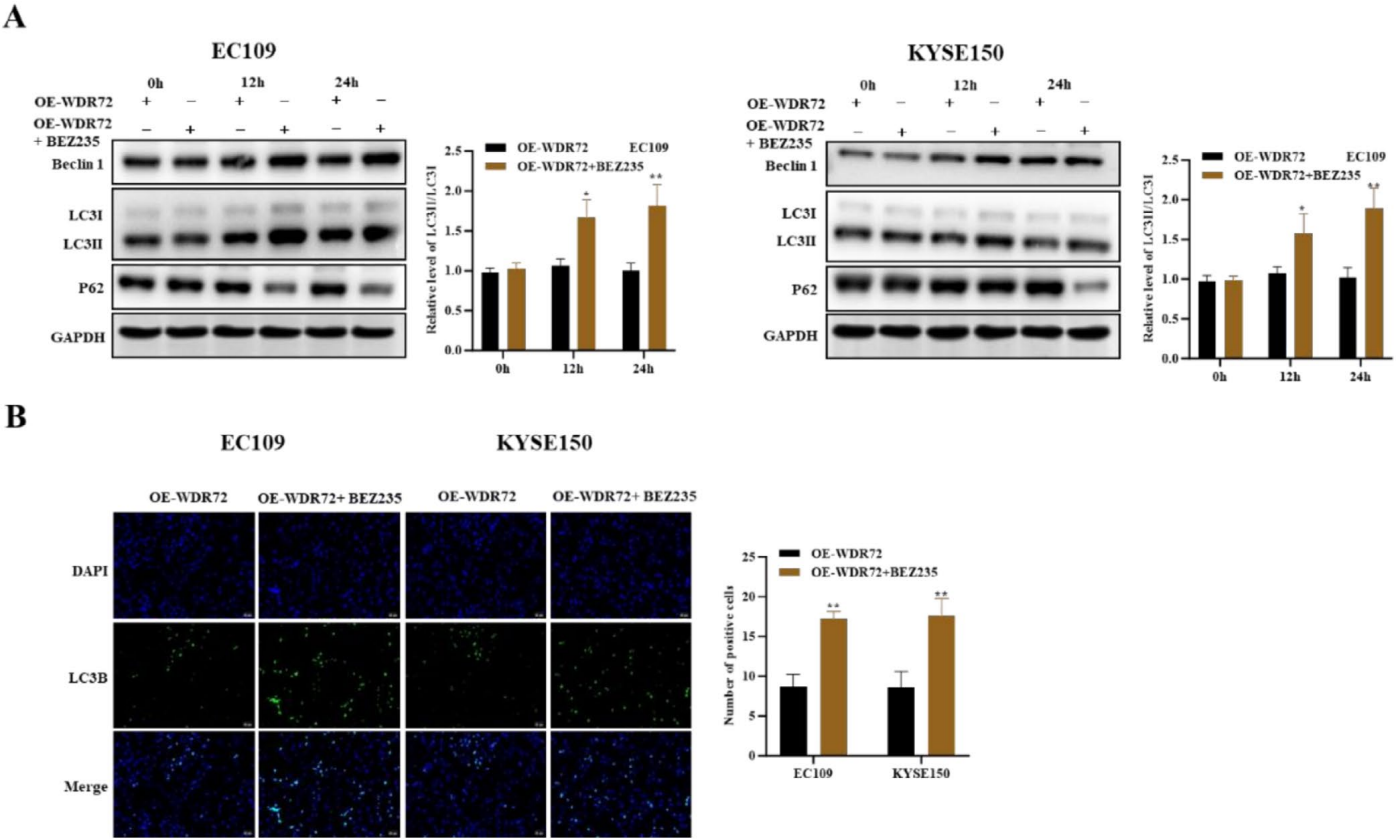
WDR72 suppresses autophagy through activation of the PI3K/Akt/mTOR pathway. (A) Western blot analysis of Beclin‐1, LC3, and p62 protein expression in EC109 and KYSE150 cells transfected with OE‐WDR72 or treated with BEZ235 in the OE‐WDR72 groups. (B) Immunofluorescence staining for LC3B puncta showing restoration of autophagy in WDR72‐overexpressing cells upon BEZ235 treatment. Data are shown as the mean ± SD of three independent experiments and analyzed using a two‐tailed unpaired t‐test. *p < 0.05, **p < 0.01.

Following the confirmation that WDR72 suppresses autophagy and activates the PI3K/Akt/mTOR pathway, we sought to determine if the autophagy suppression is mediated by this pathway. We again performed rescue experiments, treating WDR72‐overexpressing cells with the inhibitor BEZ235. Through Western blot, we observed that BEZ235 treatment reversed the effects of WDR72, leading to a notable increase in Beclin1 levels and the LC3II/LC3I ratio, while simultaneously reducing the P62 levels (Figure 5A). In addition, IF staining further proved that the reduced LC3B fluorescence level, a characteristic of WDR72 overexpression, was markedly increased by BEZ235 treatment (Figure 5B). These outcomes suggest that WDR72 suppresses cell autophagy by activating the PI3K/Akt/mTOR pathway.

### 3.6 WDR72 Depletion Suppresses ESCC Tumor Growth and Metastasis In Vivo

**FIGURE 6:**
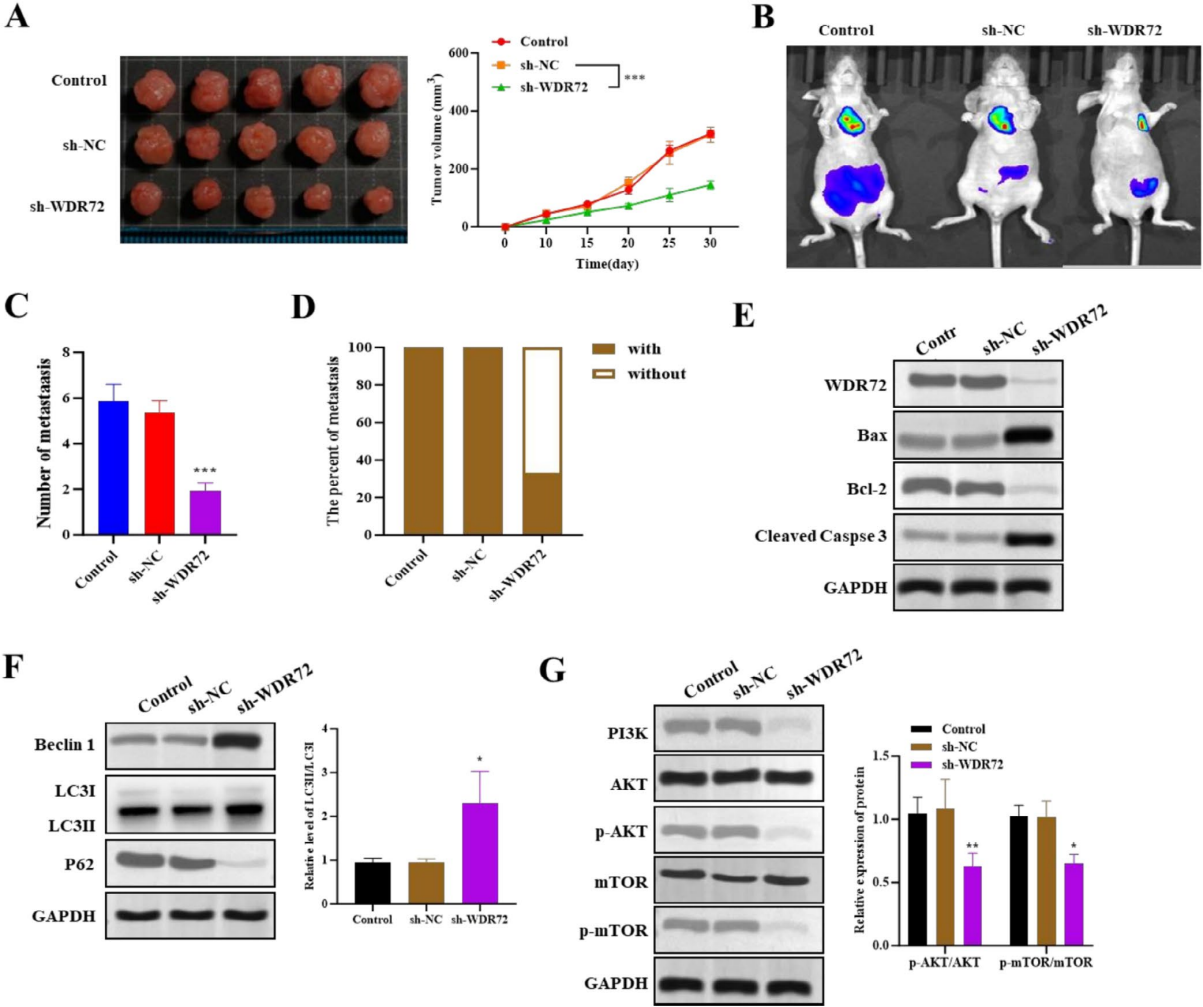
WDR72 knockdown inhibits tumor growth and metastasis in vivo by promoting apoptosis and autophagy. (A) Representative images and growth curves of xenograft tumors derived from control, sh‐NC, and sh‐WDR72 EC109 cells. Tumor volumes were measured every 5 days, and excised tumors were photographed at endpoint. (B) Representative bioluminescence images showing metastatic burden in mice injected with sh‐NC or sh‐WDR72‐transfected EC109 cells via tail vein. (C) Quantification of the number of metastatic nodules. (D) Quantitative analysis of metastatic incidence in each group. (E‐G) Western blot analysis of WDR72, apoptosis‐related proteins (Bax, Bcl‐2, cleaved caspase‐3), autophagy‐related proteins (Beclin‐1, LC3, p62), and pathway‐related proteins (PI3K, AKT, p‐AKT, mTOR, p‐mTOR) in tumor tissues. Data represent the mean ± SD (*n* = 5 per group) and were analyzed by a two‐tailed unpaired t‐test or one‐way ANOVA followed by Tukey's post hoc test. ***p < 0.001.

Finally, we conducted xenograft and metastasis assays to validate the in vitro findings and confirm the therapeutic potential of targeting WDR72 in vivo. To this end, we established xenograft and metastasis mouse models using EC109 cells stably transfected with sh‐WDR72 or sh‐NC. Compared with controls, the sh‐WDR72 group exhibited significantly reduced tumor volume and weight (Figure 6A). In the lung metastasis model, WDR72 knockdown markedly decreased bioluminescence intensity and the number of metastatic nodules, as observed by IVIS imaging (Figure 6B,C). The incidence of metastasis was also substantially lower in the sh‐WDR72 group than in controls (Figure 6D).

To confirm the mechanism in the excised tumor tissues, Western blot analysis was performed. Consistent with in vitro results, sh‐WDR72 tumors showed reduced WDR72 and anti‐apoptotic Bcl‐2 expressions, alongside elevated pro‐apoptotic Bax and cleaved caspase3 expressions (Figure 6E). Additionally, WDR72 depletion significantly enhanced Beclin1 levels and the LC3 II/LC3I ratio, while reducing P62 levels, confirming that WDR72 downregulation facilitates both apoptosis and autophagy in vivo (Figure 6F). Finally, Western blot analysis of the signaling axis demonstrated that the expressions of PI3K, p‐AKT, and p‐mTOR were significantly declined by WDR72 silencing in the tumor tissues (Figure 6G). Collectively, these in vivo results confirm that WDR72 silencing inhibits ESCC tumor growth and metastasis by promoting apoptosis and autophagy via suppression of the PI3K/Akt/mTOR pathway.

## 4 Discussion

This study identifies WDR72 as a putative oncogenic driver in ESCC and delineates a mechanistic axis whereby WDR72 promotes malignant phenotypes by activating PI3K/Akt/mTOR signaling and suppressing autophagy. We show that WDR72 is upregulated in ESCC tissues and cell lines; its knockdown diminishes proliferation, clonogenicity, migration, and invasion while increasing apoptosis in vitro, and it restrains tumor growth and lung metastasis in vivo. Mechanistically, WDR72 loss elevates Beclin‐1 and the LC3‐II/LC3‐I ratio with a concomitant decrease in p62/SQSTM1, consistent with enhanced autophagic flux, whereas WDR72 overexpression induces the opposite effects. Pharmacological blockade with the dual PI3K/mTOR inhibitor BEZ235 reverses WDR72‐driven increases in p‐AKT and p‐mTOR, restores autophagy, and abrogates the pro‐tumorigenic effects of WDR72. Together, these findings support a model in which WDR72 facilitates ESCC progression by inhibiting autophagy through activation of the PI3K/Akt/mTOR pathway.

Our results align with and extend several lines of prior evidence. Clinically, ESCC remains a highly lethal malignancy in which metastasis drives mortality [26, 27]. Consistent with the centrality of metastatic competence, we observed that WDR72 knockdown curtailed migration/invasion in vitro and reduced lung metastases in vivo, supporting a functional contribution of WDR72 to the metastatic phenotype. At the gene level, WDR72 has been linked to tumor biology in multiple settings: high expression has been reported in esophageal carcinoma at the transcriptomic level [15], and context‐dependent roles have been noted in other cancers, including pro‐stemness AKT signaling in lung cancer and prognostic associations in renal carcinoma [12, 13, 14]. Our data in ESCC are most consistent with the lung cancer paradigm; WDR72 enhances oncogenic signaling and malignant traits, and helps to resolve the previously “unknown” role of WDR72 in ESCC by providing mechanistic evidence.

The autophagy axis offers additional context. Autophagy can either constrain early tumorigenesis or sustain established tumors under stress [28, 29]. In ESCC, both pro‐ and anti‐tumor influences of autophagy have been reported (e.g., PSMD2 restrains autophagy and promotes ESCC; metformin enhances autophagy and suppresses ESCC through STAT3 inhibition) [30, 31]. Consistent with these observations, we find that WDR72 depletion augments autophagic flux (increase in Beclin‐1 and LC3‐II/LC3‐I levels, while a decrease in p62) in parallel with reduced tumor cell viability and motility, suggesting that autophagy restoration exerts a tumor‐suppressive effect in ESCC. Thus, our data place WDR72 on the side of autophagy suppression: WDR72 depletion increases Beclin‐1 and LC3‐II/LC3‐I and decreases p62, canonical indicators of augmented autophagic flux [32, 33, 34].

The PI3K/Akt/mTOR pathway is a central coordinator of growth and metabolism and a well‐recognized negative regulator of autophagy [35, 36]. Aberrant activation of this axis promotes ESCC aggressiveness and poor outcomes [36, 37]. Our data add WDR72 to the catalog of upstream modulators that converge on this pathway: WDR72 overexpression increases PI3K, p‐AKT, and p‐mTOR, while BEZ235 normalizes these signals and functionally reverses WDR72‐driven proliferation, survival, migration, and invasion. These pharmacologic “rescue” experiments strengthen the causal link between WDR72 and PI3K/Akt/mTOR activity in ESCC. Notably, prior studies in other cancers have connected WDR72 to AKT‐centered signaling and tumor microenvironmental remodeling [13, 14], whereas transcriptomic analyses in EC predicted WDR72 upregulation without establishing a mechanism [15]. By integrating expression, functional, and pathway interrogation data, including in vivo validation, our work addresses this gap and provides mechanistic evidence that WDR72 promotes ESCC through PI3K/Akt/mTOR‐dependent autophagy inhibition.

This work should also be interpreted alongside reports that therapeutically targeting PI3K/Akt/mTOR can enhance autophagy and limit tumor growth in various malignancies, including models of prostate and ovarian cancer [38, 39]. Our findings extend this therapeutic rationale to ESCC in the specific context of WDR72 dependence: when WDR72 is high, PI3K/Akt/mTOR activation suppresses autophagy and drives malignant behavior; inhibiting the pathway (with BEZ235 in our study) restores autophagy and mitigates these phenotypes. These data suggest WDR72 as a candidate biomarker for selecting patients who might benefit from PI3K/mTOR‐targeted strategies and raise the possibility that co‐targeting WDR72 signaling and PI3K/Akt/mTOR could be synergistic in ESCC.

Notwithstanding these strengths, our study has limitations. First, although BEZ235 provided pharmacological evidence for pathway involvement, complementary genetic perturbations (e.g., AKT or mTOR knockdown/constitutive mutants) would more precisely map the signaling hierarchy downstream of WDR72. Second, our primary mechanistic focus was the PI3K/Akt/mTOR axis; other WDR72‐interacting pathways (e.g., additional WD‐repeat‐mediated scaffolding networks) were not explored and may contribute to the phenotype. Third, the clinical sample size used for validation was relatively limited (*n* = 15 paired tissues). This cohort was primarily designed to provide preliminary and exploratory validation of candidate biomarkers identified through large‐scale bioinformatic screening and experimental analyses. Although consistent expression patterns were observed, the limited sample size may restrict statistical power and generalizability. Therefore, validation using larger independent clinical cohorts, ideally incorporating survival analysis and multicenter datasets, will be necessary to establish the prognostic and translational significance of WDR72. Additionally, tissue‐level validation of signaling and autophagy‐related proteins remains incomplete. Although Western blot and functional experiments confirmed the regulatory relationship between WDR72 and the PI3K/Akt/mTOR‐autophagy axis, histopathological validation of WDR72, p‐AKT, p‐mTOR, and autophagy markers in human ESCC specimens and xenograft tumor tissues was not performed due to limited availability of clinical samples. Future studies incorporating comprehensive immunohistochemical analyses will further strengthen the clinical and translational relevance of the WDR72‐mediated regulatory network in ESCC.

## 5 Conclusion

In summary, WDR72 is overexpressed in ESCC and functions as a tumor promoter by activating the PI3K/Akt/mTOR pathway to suppress autophagy, thereby enhancing proliferation, survival, migration, and invasion in vitro and accelerating tumor growth and metastasis in vivo. Genetic depletion of WDR72 or pharmacologic blockade of PI3K/mTOR reverses these effects. These findings provide mechanistic insight into ESCC pathogenesis and suggest WDR72 as a potential biomarker and therapeutic target, warranting further translational investigation.

## Ethics Statement

All the experimental procedures involving animals were conducted following ARRIVE guidelines and were approved by the Ethics Committee of the First Affiliated Hospital of Chongqing Medical University. The study involving human subjects complied with the Declaration of Helsinki and was approved by the Ethics Committee of the First Affiliated Hospital of Chongqing Medical University. All participants provided written informed consent.

## Conflicts of Interest

The authors declare no conflicts of interest.

## Supporting information


**Figure S1:** Chloroquine reverses the effects of sh‐WDR72. (A) Western blot analysis of Beclin‐1, LC3, and p62 protein expression in EC109 cells transfected with sh‐WDR72 and treated with/without chloroquine for 24 h. (B) Immunofluorescence staining for LC3B puncta showing restoration of autophagy in WDR72 silent cells upon chloroquine treatment. Data are shown as the mean ± SD of three independent experiments and analyzed using a two‐tailed unpaired *t*‐test. ***p < 0.001.
**Figure S2:** AKT knockdown reverses the effects of WDR72. (A) GEPIA database shows a significant positive expression correlation between WDR72 and PIK3CA in ESCC. (B) Western blot analysis of Beclin‐1, LC3, and p62 protein expression in EC109 and KYSE150 cells under indicated transfections. (C) Immunofluorescence staining for LC3B in EC109 and KYSE150 cells under indicated transfections. Data are shown as the mean ± SD of three independent experiments and analyzed using a two‐tailed unpaired *t*‐test. **p < 0.01, ***p < 0.001.

## Data Availability

The data that support the findings of this study are available from the corresponding author upon reasonable request.
