# Supplementary material for: WDR72 Drives Esophageal Squamous Cell Carcinoma Progression by Inhibiting Autophagy via the PI3K/Akt/mTOR Pathway
Source: Kaohsiung J Med Sci. 2026 Jun 1:e70235. Online ahead of print. doi: 10.1002/kjm2.70235 (PMC13399781; doi:10.1002/kjm2.70235)
Supplement: Supplementary file 1 — Figure S1: Chloroquine reverses the effects of sh‐WDR72. (A) Western blot analysis of Beclin‐1, LC3, and p62 protein expression in EC109 cells transfected with sh‐WDR72 and treated with/without chloroquine for 24 h. (B) Immunofluorescence staining for LC3B puncta showing restoration of autophagy in WDR72 silent cells upon chloroquine treatment. Data are shown as the mean ± SD of three independent experiments and analyzed using a two‐tailed unpaired t‐test. ***p < 0.001. Figure S2: AKT knockdown reverses the effects of WDR72. (A) GEPIA database shows a significant positive expression correlation between WDR72 and PIK3CA in ESCC. (B) Western blot analysis of Beclin‐1, LC3, and p62 protein expression in EC109 and KYSE150 cells under indicated transfections. (C) Immunofluorescence staining for LC3B in EC109 and KYSE150 cells under indicated transfections. Data are shown as the mean ± SD of three independent experiments and analyzed using a two‐tailed unpaired t‐test. **p < 0.01, ***p < 0.001. [file KJM2-9999-e70235-s001.docx]

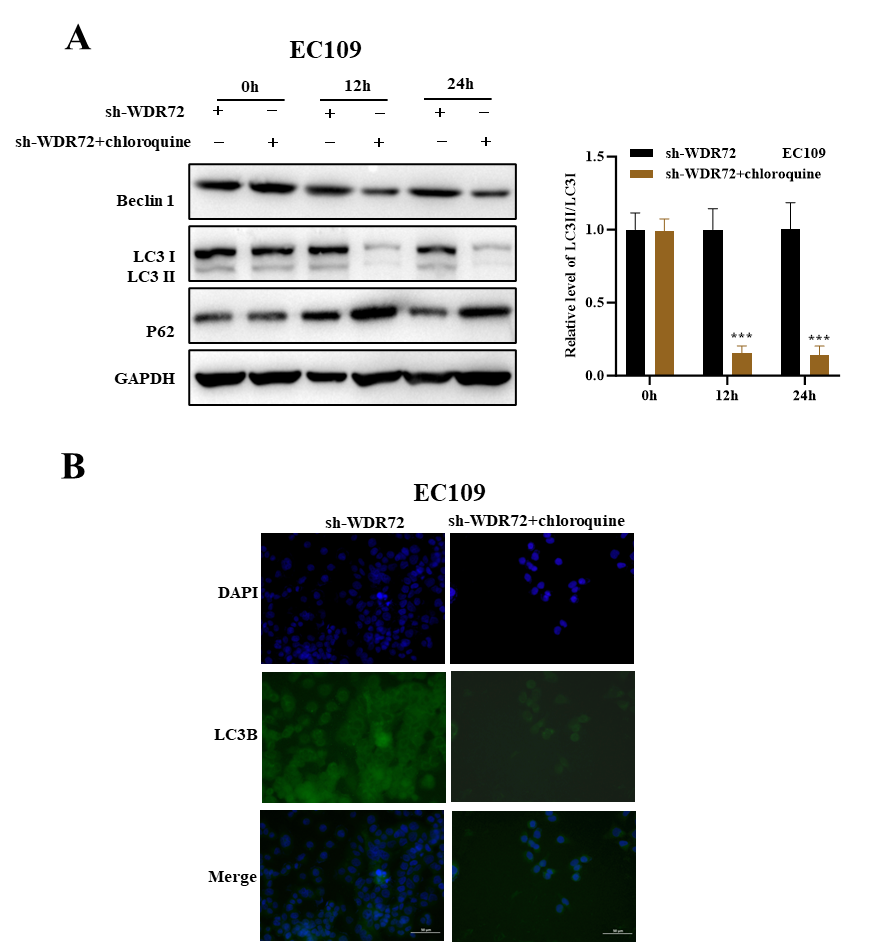


**Supplementary Figure 1.** **Chloroquine reverses the effects of sh-WDR72.** (A) Western blot analysis of Beclin-1, LC3, and p62 protein expression in EC109 cells transfected with sh-WDR72 and treated with/without chloroquine for 24 hours. (B) Immunofluorescence staining for LC3B puncta showing restoration of autophagy in WDR72 silent cells upon chloroquine treatment. Data are shown as the mean ± SD of three independent experiments and analyzed using a two-tailed unpaired t-test. ^***^*p*<0.001.


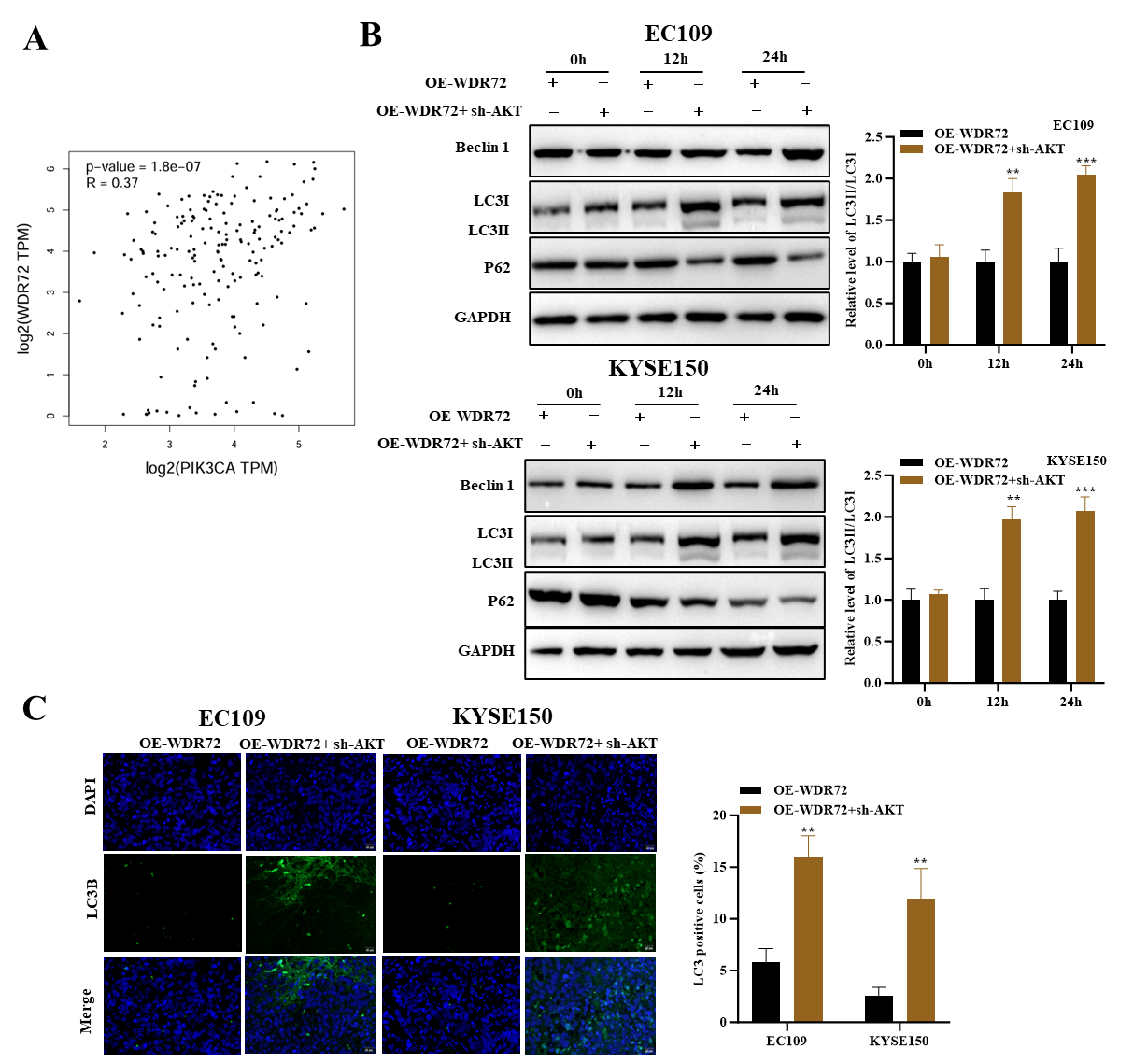


**Supplementary Figure 2.** **AKT knockdown reverses the effects of WDR72.** (A) GEPIA database shows a significant positive expression correlation between WDR72 and PIK3CA in ESCC. (B) Western blot analysis of Beclin-1, LC3, and p62 protein expression in EC109 and KYSE150 cells under indicated transfections. (C) Immunofluorescence staining for LC3B in EC109 and KYSE150 cells under indicated transfections. Data are shown as the mean ± SD of three independent experiments and analyzed using a two-tailed unpaired t-test. ^**^*p*<0.01, ^***^*p*<0.001.
